# Neural and cognitive dynamics leading to the formation of strong memories: A meta-analysis and the SAM model

**DOI:** 10.1162/imag_a_00098

**Published:** 2024-02-22

**Authors:** Hongkeun Kim

**Affiliations:** Department of Rehabilitation Psychology, Daegu University, Gyeongsan-si, Republic of Korea

**Keywords:** fMRI, episodic memory, encoding, subsequent memory, attention, hippocampus

## Abstract

The subsequent memory paradigm is a fundamental tool in neuroimaging investigations of encoding processes. Although some studies have contrasted remembered trials with forgotten ones, others have focused on strongly remembered trials versus forgotten ones. This study employed a meta-analytic approach to juxtapose the effects observed in the two types of contrast. Three distinct perspectives on memory formation—semantic elaboration, attentional focus, and hippocampal processing—yield diverse hypotheses about the regions responsible for the formation of strong memories. The meta-analysis yielded evidence supporting the attentional and semantic hypotheses while failing to substantiate the hippocampal hypothesis. The discussion section integrates these varied perspectives into a coherent view, culminating in the proposal of a model called the Significance-driven and Attention-driven Memory (SAM). Several pivotal postulates underpin the SAM model. First, it establishes a link between fluctuations in the trial-to-trial encoding performance and continuous variations in sustained attention. Second, the model contends that attention exerts a potent influence on both perceptual and semantic processing, while its impact on hippocampal processing remains moderate. Lastly, the model accentuates the heightened role of the hippocampus in significance-driven encoding, as opposed to attention-driven encoding. From a specific perspective, the model’s value lies in promoting a holistic understanding of the current extensive meta-analytic results. In a more comprehensive context, the model introduces an integrated framework that synthesizes various encoding-related cognitive and neural processes into a cohesive and unified perspective.

## 1 Introduction

### 1.1 Study purpose

Since the late 1990s (Brewer et al., 1998; Wagner et al., 1998), the subsequent memory paradigm has become a fundamental tool in functional magnetic resonance imaging (fMRI) studies of memory encoding processes. This procedure involves categorizing learning trials (i.e., events) into two groups: later remembered and forgotten, based on the participants’ performance on a later memory test. Subsequently, a direct comparison is conducted between the neural activity associated with remembered and forgotten trials. Neural regions exhibiting higher activity during the remembered trials, compared to the forgotten trials, are believed to play an essential role in successfully encoding incoming information. In the present study, the contrast of remembered > forgotten trials will be referred to as the *general-subsequent memory* (SM) contrast. This term differentiates it from another type of subsequent memory contrast discussed below and emphasizes that its target condition includes all trials successfully remembered, regardless of the confidence level.

As the subsequent memory paradigm has evolved over the years, it has given rise to several variations of the general-SM contrast. One such variation, termed the *strong-SM* contrast, has gained significant popularity. In experiments using this contrast, the participants are asked to discriminate between strong and weak memories subjectively. For example, response choices may include options like “Sure old,” “Unsure old,” and “New” (e.g., Buckner et al., 2001; Kim & Cabeza, 2007; Park et al., 2013). In experiments distinguishing between recollection and familiarity, response options might involve “Remember,” “Know,” and “New” responses (e.g., Otten, 2007; Rizio & Dennis, 2013; Uncapher & Rugg, 2008). These experiments compared the neural activity associated with remembered trials with a high degree of confidence (or recollected) and those forgotten. Compared to the general-SM contrast, the primary differentiation in the strong-SM contrast resides in incorporating solely strongly remembered trials within the target condition, excluding weakly remembered ones. The prevalent adoption of the strong-SM contrast relies on the perspective that it exhibits greater sensitivity to encoding-related neural activity compared to the general-SM contrast.

This study compared the neural effects associated with the strong-SM contrast and the general-SM contrast. This comparison was driven by the need to critically assess the assumption that encoding-related neural activity is captured more efficiently by the strong-SM contrast than by the general-SM contrast. This study aimed to identify regions with more pronounced effects from the strong-SM contrast than the general-SM contrast. Furthermore, this study sought to pinpoint regions that deviate from this pattern, potentially confirming or challenging the assumption. Certain regions associated with encoding may undertake distinct and specialized functions in shaping robust memories, differing from their role in weaker memory formation. As a result, these regions could display heightened activity levels during strongly remembered trials, in contrast to their involvement in weakly remembered ones. This elevated activity will be demonstrated through the prominence of the strong-SM effects than general-SM effects. By contrast, regions not directly implicated in establishing robust memories might show a less conspicuous correlation with the strong-SM effects. Therefore, it is possible to gain insights into the neural activity responsible for shaping strong memories by juxtaposing the strong-SM and general-SM effects.

A potential challenge in comparing the strong-SM and general-SM effects needs discussion. It is noteworthy that signals contributing to the formation of robust memories are not exclusive to the strong-SM contrast but are also encompassed within the general-SM contrast, resulting in an overlap during comparison. Importantly, it should be emphasized that this partial overlap is more likely to diminish rather than obliterate the distinctions between the two effects concerning both strong and weak memory formation. Moreover, the comparison between the strong-SM and general-SM contrasts holds practical significance despite the observed overlap, as both contrasts are widely used in subsequent memory studies.

In light of the rapid accumulation of functional neuroimaging data, the significance of meta-analyses in unveiling consistent patterns and discrepancies between studies has grown significantly. Accordingly, this study adopted a meta-analytic approach to compare the neural effects associated with the strong-SM contrast and the general-SM contrast. Prior meta-analyses have not focused on examining these two types of contrast separately, making the current study a novel contribution with previously unreported data.

### 1.2 Three divergent perspectives on memory formation

Which specific neural regions influence the development of strong memories? This study highlights three well-established and distinct viewpoints concerning memory encoding. By extracting insights from these perspectives, the study formulates three separate hypotheses about the neural processes that contribute to the formation of robust memories. The subsequent paragraphs introduce these hypotheses, laying the groundwork for interpreting the upcoming meta-analytical findings.

First, stemming from the renowned case of patient H. M. (Scoville & Milner, 1957), a widely accepted consensus has arisen, underscoring the pivotal role of the hippocampus in memory encoding (Eichenbaum, 2013; Squire & Wixted, 2011). Expanding upon this viewpoint, one can postulate that establishing robust memories hinges on heightened hippocampal processing. Approximately 10 studies supplied relevant data for investigating the hippocampal hypothesis and the subsequent two hypotheses. These studies either correlated the encoding activity with the subsequent memory strength or compared the encoding activity associated with subsequent-Remember trials versus subsequent-Know trials. Only one study (Dennis et al., 2007) reported significant hippocampal engagement using an identical threshold level across the medial temporal lobe (MTL) and non-MTL regions. In the remaining studies, the hippocampal effect either did not reach statistical significance (François et al., 2018; Gottlieb & Rugg, 2011; Henson et al., 1999; Kim & Cabeza, 2007) or became noticeable only when employing a less stringent statistical threshold, such as small volume correction (SVC), as opposed to the findings from whole-brain analysis (Kirwan et al., 2008; Qin et al., 2011; Shrager et al., 2008; Song, Jeneson, et al. 2011; Song, Wixted, et al., 2011). Together, these nuanced findings suggest the presence of a subtle effect size, a concept that will be explored in greater depth.

Second, the level-of-processing framework (Craik & Lockhart, 1972) posits that semantic elaboration, or deeper processing, enhances memory formation. A wealth of behavioral evidence supports this hypothesis (for a review, see Craik, 2002). Functional neuroimaging studies have underscored the crucial role played by the inferior prefrontal cortex (PFC) in the controlled semantic processing of incoming information (Noonan et al., 2013; Nozari & Thompson-Schill, 2016; Poldrack et al., 1999). For example, in a recent meta-analysis (Kim, 2022), neural activity was compared during the semantic processing of words (e.g., concrete/abstract judgment) and their structural processing (e.g., uppercase/lowercase judgment). The analysis revealed heightened activity during semantic processing, primarily within the left inferior frontal cortex. Drawing from this body of evidence, it is conceivable that establishing robust memories entails heightened activity within the inferior PFC. The majority of pertinent previous studies (Dennis et al., 2007; François et al., 2018; Gottlieb & Rugg, 2011; Henson et al., 1999; Kim & Cabeza, 2007; Qin et al., 2011; Shrager et al., 2008; Song, Wixted, et al., 2011) revealed the involvement of the inferior PFC, substantiating the hypothesis.

Lastly, scientific research and anecdotal evidence indicate that focused attention to incoming information enhances its registration into memory (Chun & Johnson, 2011; Craik et al., 1996; Fernandes & Moscovitch, 2000; Long et al., 2018). This evidence underpins the hypothesis that forming strong memories demands heightened concentration on incoming information. An intrinsic connectivity network, known as the dorsal attention network, is recognized widely for its pivotal role in guiding purposeful attention to incoming information from the external environment (Corbetta & Shulman, 2002; Shulman & Corbetta, 2012). Consequently, it can be conjectured that the establishment of robust memories involves escalated activity within the regions comprising the dorsal attention network. Several relevant earlier studies (Dennis et al., 2007; Kim & Cabeza, 2007; Kirwan et al., 2008; Qin et al., 2011; Song, Jeneson, et al., 2011) revealed significant activation of specific areas within the dorsal attention network, reinforcing the hypothesis. These regions encompass the inferior frontal junction (located at the intersection of the inferior frontal sulcus and the precentral sulcus), the mid-fusiform gyrus, and the dorsal parietal cortex.

After summarizing the three distinct viewpoints and their corresponding hypotheses, it is essential to emphasize that these perspectives are not mutually exclusive. Each one illuminates a distinct brain region or specific network in conjunction with its associated cognitive function. In particular, the hippocampal, semantic, and attentional viewpoints underscore a distinct role of the hippocampus, inferior PFC, and dorsal attention network in shaping robust memories, respectively. The Discussion section combines these different viewpoints into a harmonious structure, aiming to cultivate a holistic comprehension of the current extensive meta-analytic results. This effort culminates in a streamlined model of encoding processes, highlighting the neural and cognitive dynamics that lead to the establishment of memories.

## 2 Methods

### 2.1 Data collection

The literature search, paper selection, and analysis generally conformed to the neuroimaging meta-analysis guidelines proposed by Müller et al. (2018). The PubMed Database was searched comprehensively in February 2023 to identify suitable studies for inclusion in the meta-analysis. The search query utilized was fMRI AND (“subsequent memory” OR “episodic memory encoding”). This initial search was complemented by applying a similar method to the Google Scholar database and manually reviewing the reference lists of selected papers. The retrieved studies were screened meticulously, retaining only those studies/experiments (where an “experiment” refers to an individual contrast reported in each study) that adhered to the following inclusion and exclusion criteria:

1. Only studies that examined the strong-SM contrast or the general-SM contrast were included.
2. Only studies involving neurologically healthy participants were included. Studies comparing clinical patients with healthy controls were incorporated, provided that results for the control group were reported separately.
3. Only studies that reported their results in either Talairach or Montreal Neurological Institute (MNI) space were included.
4. Some studies included two or more pertinent experiments. To maintain a balanced representation, only one experiment was selected from each of these studies. Specifically, the experiment chosen was the one with the highest number of peak activation foci. This selection was made with the expectation of augmenting the sensitivity of the meta-analysis.
5. Studies relying exclusively on predefined regions of interest (ROI) were omitted from consideration. This exclusion was motivated by the commitment to maintaining an equal a priori activation probability for each voxel, a crucial principle for coordinate-based meta-analyses. In instances where studies encompassed both whole-brain analyses and analyses involving ROI or SVC, peaks derived from ROI/SVC were included only if they met the same statistical criterion applied to the rest of the brain.
6. Experiments utilizing emotional or reward-related stimuli were excluded from consideration. The author’s recent review (Kim, 2022) of the pertinent literature underscored that studies employing such stimuli often yield subsequent memory effects that diverge from those observed with more commonplace stimuli. Consequently, this study sought to ensure a more focused and coherent analysis by deliberately excluding experiments involving emotional or reward-related stimuli.
7. When selecting experiments utilizing the strong-SM contrast, the following two types of contrast were included: (a) confidently remembered > forgotten and (b) recollected > forgotten. The rationale for incorporating (b) is that, even though there is no one-to-one direct correspondence, "Remember" and "Know" responses generally align with high- and low-confidence recognition, respectively (Dunn, 2004; J. T. Wixted et al., 2010).

Ultimately, 70 research papers satisfied the criteria for inclusion in the meta-analysis. More details regarding these studies, including a tabulated overview of all encompassed experiments and a comprehensive reference list, can be accessed in the online Supplementary Material accompanying this article (refer to Tables S1 and S2 and Supplementary References).

### 2.2 Subsample characteristics

The strong-SM subsample comprised 31 experiments gathered from 29 distinct studies (discrepancies in counts arose from one study including three independent samples, each with its own results), collectively contributing 354 peak foci. This array of experimental inquiries involved a collective total of 853 participants, comprising young adults in 23 experiments, older adults in 4, a combination of young and older adults in 3, and a mix of young, middle-aged, and older adults in 1. The stimuli took various forms within this set of experiments: words in 15 instances, scenes in 9, objects in 2, faces in 2, abstract shapes in 1, and combinations, such as faces and scenes, in 2 cases. The retrieval task/subsequent memory contrast in these experiments comprised two types. In 25 experiments, memory confidence ratings were employed, focusing on the contrast between confidently remembered and forgotten trials. Additionally, the Remember-Know procedure was applied in six experiments, specifically comparing recollected trials to forgotten trials.

The general-SM subsample comprised 36 experiments from 36 studies, collectively contributing 259 peak foci. A total of 807 individuals participated in these experiments, including young adults in 31 experiments, older adults in 2, middle-aged adults in 1, and a combination of young and older adults in 2. The stimuli presented to participants encompassed words in 18 experiments, scenes in 7, objects in 6, faces in 3, and hybrid stimuli in 2 instances. The retrieval task/subsequent memory contrast in these experiments consisted of three types. First, the old/new recognition task was applied in 23 experiments, focusing on the contrast between remembered and forgotten trials. Second, memory confidence ratings were employed in seven experiments, where the selected contrast compared subsequently remembered trials (collapsed over high- and low-confidence) against their forgotten counterparts. Third, item/source retrieval tasks were utilized in six experiments. The chosen contrast compared trials in which the items were subsequently recognized correctly, irrespective of whether source information was correctly recalled, against trials in which they were subsequently forgotten.

Since the main hypotheses of the present study concern the comparison of the strong-SM and general-SM effects, it was important to match the two subsamples in nuisance variables to the greatest extent possible. First, according to the power analysis of the Activation Likelihood Estimation (ALE) meta-analysis (Eickhoff et al., 2016), a minimum of 17 experiments was recommended to ensure the detection of moderate effects. Both subsamples in this study met this recommended criterion. Second, the proportion of experiments using verbal stimuli showed similarity between the two subsamples: 45.2% in the strong-SM and 50.0% in the general-SM, with no significant statistical difference (χ² < 1). Third, the proportion of experiments with young adults only was similar between the two subsamples: 74.2% in the strong-SM and 86.1% in the general-SM, with no significant statistical difference (χ² = 1.513, p = 0.219). Consequently, none of the variations observed in the primary analyses between the two subsamples can be attributed to a disparity in the inclusion of verbal versus pictorial stimulus types or in the age of participants. Despite this balance between the two subsamples in certain crucial variables, it is worth noting that they may still differ in other variables, necessitating a careful interpretation of the main results.

### 2.3 Meta-analysis

All meta-analyses were conducted using the ALE algorithm (Eickhoff et al., 2009, 2011) through GingerALE software version 3.0.2 (http://www.brainmap.org/ale). This algorithm evaluates the spatial convergence of activation foci across independent studies. To maintain consistency in coordinate space, all activation coordinates originally reported in Talairach space were transformed into MNI space using the GingerALE transformation tool prior to analysis. The ALE method was applied to analyze the current data, using the following steps (the author discussed analogous methodological steps in Kim, 2023 and other studies).

Separate meta-analyses were performed to evaluate the strong-SM effects and the general-SM effects. Each focus gathered from the selected studies was modeled as the center point of a 3-D Gaussian probability distribution, addressing the inherent spatial uncertainty linked to each focus. An extended ALE algorithm weighed the number of participants in each study to determine the full width at half maximum (FWHM) of the Gaussian distribution. For example, within the meta-analysis of the strong-SM effects, the FWHM values ranged from 8.50 mm to 9.66 mm, with a median of 9.33 mm. The 3-D Gaussian probabilities were aggregated across all reported foci within a given study and, subsequently, across all studies included in the meta-analysis, yielding voxel-wise ALE scores. These scores were tested against an analytically derived null distribution using a cluster-level familywise error (FWE) of *p* < 0.05 and a cluster-forming voxel-level threshold of *p* < 0.005. For the MTL region, clusters that did not survive the FWE-correction, but exceeded a spatial extent threshold of 100 mm^3^ were also reported to allow more sensitive analyses in this critical memory region.

Contrast analysis was conducted across their respective ALE maps to investigate the differences between the strong-SM and general-SM effects. All studies corresponding to these effects were aggregated and then randomly divided into two groups, mirroring the original sizes. Voxel-wise ALE scores were calculated for each group, and their differences were calculated through subtraction. This procedure was repeated 10,000 times, culminating in the establishment of an empirical null distribution encompassing ALE-score differences between the two effects. The disparities observed in the ALE scores underwent testing against this null distribution, employing a voxel-wise threshold of *P* > 0.95 (95% probability for a true difference) with a cluster size threshold of 500 mm^3^. These threshold levels align with those previously used in analogous ALE contrast analyses (e.g., Rodríguez-Nieto et al., 2022; Rottschy et al., 2012). Keeping with the single-study meta-analyses, a cluster size threshold of 100 mm³ was applied specifically to the MTL region.

The meta-analysis outcomes were visualized by superimposing the thresholded ALE maps onto the inflated surface of a population-average, landmark-, and surface-based (PALS) atlas using the Caret software suite (Van Essen, 2005). The thresholded maps were also projected onto the International Consortium for Brain Mapping template using Mango software (http://ric.uthscsa.edu/mango) to ensure the visibility of hippocampal results not discernible on the PALS atlas.

### 2.4 Analysis of association with intrinsic networks

As delineated in the Introduction, one of the present hypotheses posits a more robust association of the dorsal attention network with the strong-SM effects compared with the general-SM effects. To assess this hypothesis, the study measured the strength of association between a specific ALE map and an intrinsic network. The techniques employed for this measurement were as follows. Yeo et al. (2011) partitioned the cerebral cortex into seven distinct intrinsic networks based on an analysis of resting-state functional connectivity from a large-scale dataset comprising 1000 participants. These seven networks are the dorsal attention network, default mode network, frontoparietal control network, ventral attention network, limbic network, visual network, and somatomotor network. This seven-network model was chosen to estimate the boundaries of the intrinsic networks. This selection was based on its demonstrated utility in previous studies (e.g., Benoit & Schacter, 2015; Fox et al., 2015; Kim, 2021) as a reference frame for interpreting the meta-analysis maps.

Nevertheless, intrinsic network boundaries differ according to the task being performed (Krienen et al., 2014) and the estimates of these boundaries only loosely align across different analytical methods (e.g., Eickhoff et al., 2018; Power et al., 2011). Therefore, the Yeo seven-network model was used as a helpful reference rather than a fixed template. The association strength between a specific ALE map and an intrinsic network was measured through a ratio coefficient. This coefficient was calculated by dividing the overlapping area between the ALE map and the intrinsic network by the total area of the network. By accounting for the diverse sizes of different networks, the ratio coefficient offered a standardized metric for quantifying the association.

## 3 Results

### 3.1 Non-MTL regions

**Fig. 1.**
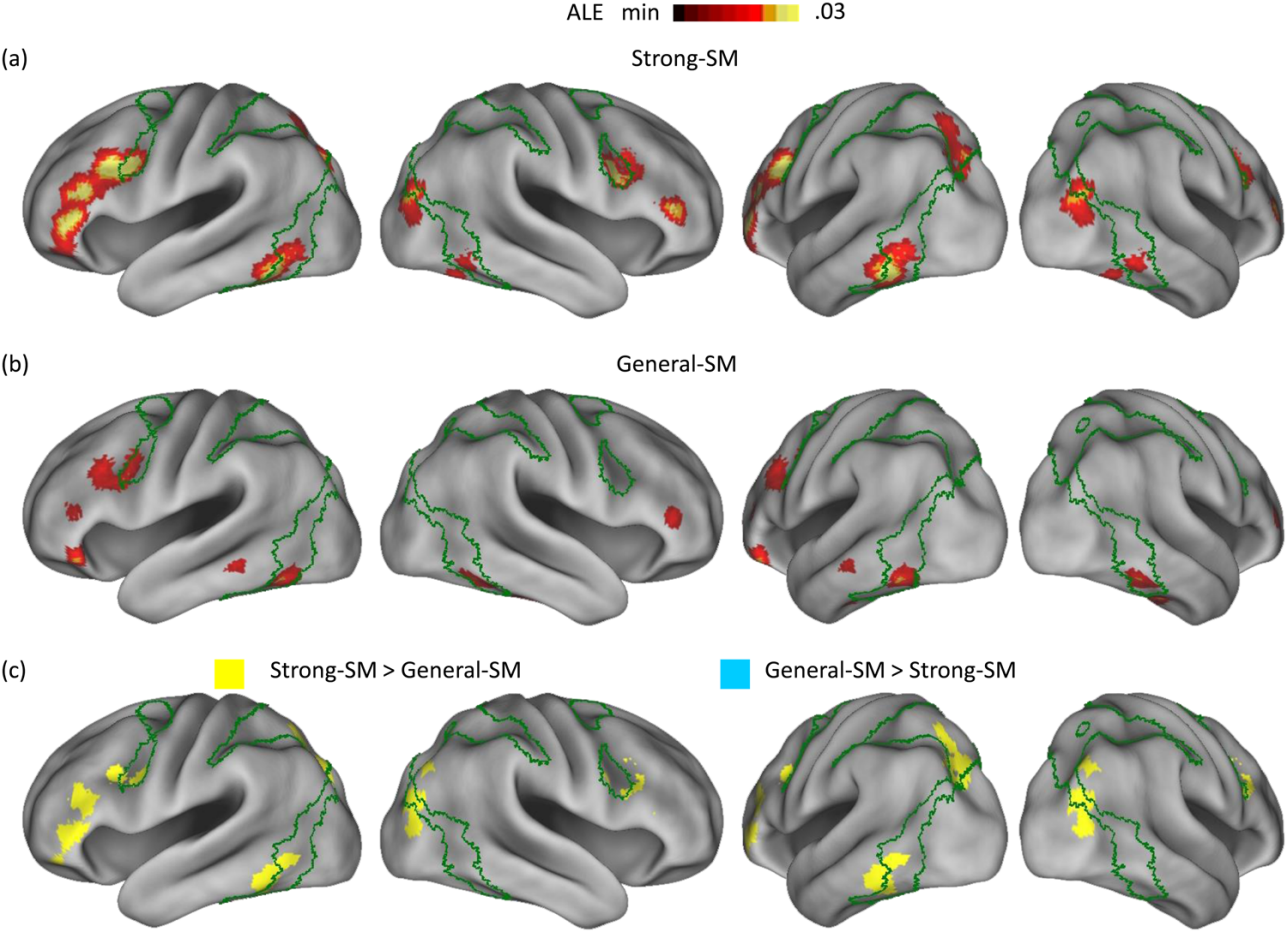
Non-medial temporal lobe findings: Part (a) displays the significant regions identified in the ALE meta-analysis of the strong-SM effects. Part (b) illustrates the corresponding regions for the general-SM effects. Part (c) depicts the significant regions arising from directly comparing these two effects. The green lines represent the estimated boundaries of the dorsal attention network in Yeo et al.’s (2011) seven-network model.

For clarity and focused discussion, the findings for non-MTL and MTL regions will be presented separately. Table 1 and Figure 1 show the outcomes for non-MTL regions. The strong-SM effects (strongly remembered > forgotten) were observed in various regions, including the bilateral inferior PFC, bilateral inferior frontal junction, left posterior superior parietal lobe (SPL)/intraparietal sulcus (IPS), bilateral mid-fusiform gyrus, and right middle occipital gyrus (see Fig. 1a). Furthermore, the general-SM effects (remembered > forgotten) were identified in the bilateral inferior PFC, left inferior frontal junction, left middle temporal gyrus, and bilateral mid-fusiform gyrus regions (Fig. 1b).

**Table 1.** Non-medial temporal lobe findings: Separate meta-analyses of the strong-SM and general-SM effects and a comparison of these two effects.

Lobe	Volume (mm^3^)	MNI			ALE	Z	Region
		*x*	*y*	*z*			
*Strong-SM*							
Frontal	16200	-42	6	28	0.037		Left inferior PFC and IFJ
	3632	46	6	28	0.041		Right IFJ
	2504	48	34	14	0.033		Right inferior PFC
Parietal	3832	-26	-76	38	0.028		Left posterior SPL/IPS
Temporal	5408	-48	-54	-16	0.046		Left mid-fusiform gyrus
	1680	50	-58	-12	0.020		Right mid-fusiform gyrus
Occipital	2072	42	-80	18	0.028		Right middle occipital gyrus
*General-SM*							
Frontal	4256	-46	8	30	0.024		Left IFJ
	1576	46	32	12	0.018		Right inferior PFC
	1568	-44	28	-8	0.022		Left inferior PFC
	1488	-48	34	14	0.025		Left inferior PFC
Temporal	3536	-44	-56	-12	0.027		Left mid-fusiform gyrus and MTG
	2120	50	-52	-12	0.022		Right mid-fusiform gyrus
*Strong-SM > General-SM*							
Frontal	4648	-42	30	6		3.72	Left inferior PFC
	1440	46	16	26		3.19	Right IFJ
	1416	-36	8	30		2.52	Left IFJ
	680	56	30	14		2.68	Right inferior PFC
	624	-48	7	47		2.58	Left posterior MFG
	536	-54	12	40		2.21	Left posterior MFG
Parietal	2728	-31	-75	35		3.43	Left posterior SPL/IPS
	664	34	-76	36		2.63	Right posterior IPS
Temporal	2768	-56	-58	-11		3.89	Left mid-fusiform gyrus
Occipital	1624	41	-79	21		2.50	Right middle occipital gyrus
*General-SM > Strong-SM*							
(None)							

ALE, activation likelihood estimation; IFJ, inferior frontal junction; IPS, intraparietal sulcus; MFG, middle frontal gyrus; MTG, middle temporal gyrus; PFC, prefrontal cortex; SM, subsequent memory; SPL, superior parietal lobe.

After a direct comparison of the two effects, it was observed that the bilateral inferior PFC (particularly the left side), bilateral inferior frontal junction, left posterior middle frontal gyrus, left posterior SPL/IPS, right posterior IPS, left mid-fusiform gyrus, and right middle occipital gyrus regions exhibited a more robust association with the strong-SM effects than the general-SM effects (depicted as yellow regions in Fig. 1c). Conversely, no regions exhibited the opposite pattern, indicating a stronger association with the general-SM effects than the strong-SM effects.

**Fig. 2.**
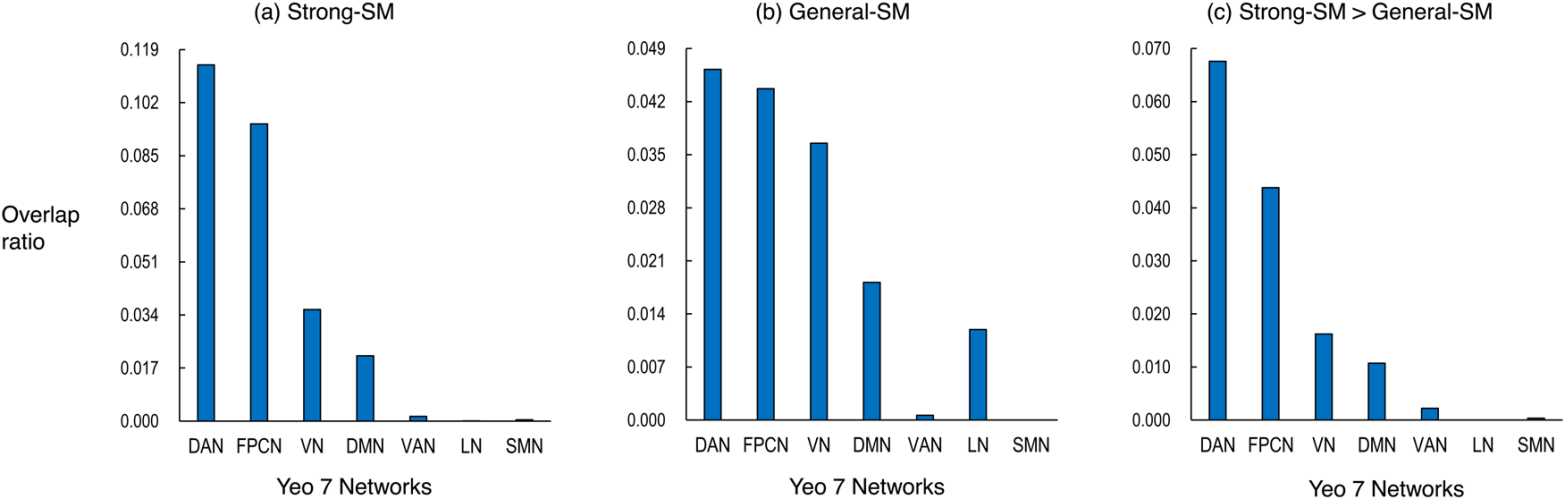
Part (a) displays the degree of association between the Yeo 7-networks and the regions showing the strong-SM effects. Part (b) displays the corresponding association for the regions exhibiting the general-SM effects. Part (c) illustrates the corresponding association for the regions where the strong-SM effects are more pronounced than the general-SM effects. Each bar in the figure represents the calculated value obtained by dividing the overlapping area between the network and the respective effect by the total area of the network. DAN, dorsal attention network; DMN, default mode network; FPCN, frontoparietal control network; LN, limbic network; SMN, somatomotor network; VAN, ventral attention network; VN, visual network.

The normalized overlap index, detailed in the Method section, was computed to evaluate the association strength of the strong-SM and general-SM effects with the Yeo seven networks. Results are presented in Figure 2. Regions linked to the strong-SM effects demonstrated the strongest alignment with the dorsal attention network, followed by the frontoparietal control network (Fig. 2a). This alignment with the dorsal attention network was substantiated by the involvement in the bilateral inferior frontal junction, left posterior SPL/IPS, bilateral mid-fusiform gyrus, and right middle occipital gyrus regions (refer to significant voxels within or adjacent to the green border in Fig. 1a). The connection to the frontoparietal control network was predominantly established through the engagement of the left inferior PFC regions.

The areas linked to the general-SM effects demonstrated the highest correspondence with the dorsal attention network, with the frontoparietal control network following closely (Fig. 2b). The connection between the general-SM effects and the dorsal attention network involved the participation of regions such as the left inferior frontal junction and bilateral mid-fusiform gyrus (Fig. 1b). The association with the frontoparietal control network was primarily established through the engagement of the bilateral inferior PFC regions.

A consistent trend emerged when calculating the normalized overlap index for regions exhibiting a stronger association with the strong-SM effects compared to the general-SM effects. These regions primarily aligned with the dorsal attention network, followed by the frontoparietal control network (Fig. 2c). Alignment with the dorsal attention network involved the bilateral inferior frontal junction, left posterior SPL/IPS, left mid-fusiform gyrus, and right middle occipital gyrus regions (Fig. 1c). Affiliation with the frontoparietal control network was linked to the engagement of the bilateral inferior PFC regions, particularly on the left side.

### 3.2 MTL regions

**Fig. 3.**
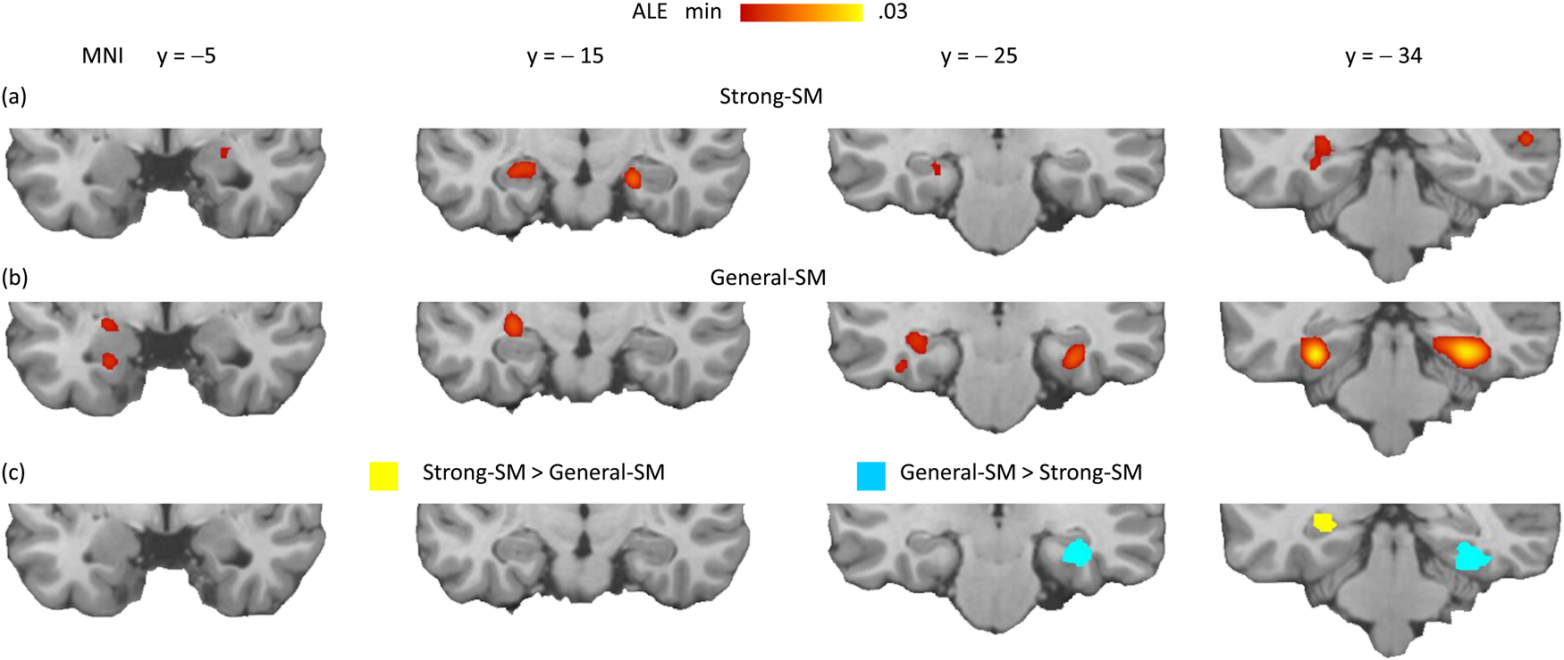
Medial temporal lobe findings: part (a) displays the significant regions identified in the ALE meta-analysis of the strong-SM effects. Part (b) illustrates the corresponding regions for the general-SM effects. Part (c) shows the significant regions that arise from directly comparing these two effects.

Table 2 and Figure 3 present the outcomes in the MTL. The strong-SM effects were observed in the bilateral hippocampus, bilateral amygdala, and bilateral parahippocampal gyrus regions (Fig. 3a). The general-SM effects encompassed the bilateral hippocampus, bilateral parahippocampal gyrus, and left amygdala regions (Fig. 3b). A comparison of the two effects revealed that a section of the left posterior hippocampus/parahippocampal gyrus exhibited a stronger association with the strong-SM effects than the general-SM effects (indicated by yellow regions in Fig. 3c). Conversely, a segment of the right posterior hippocampus/parahippocampal gyrus showed the opposite pattern, indicating a stronger association with the general-SM effects than the strong-SM effects (cyan regions).

**Table 2.** Medial temporal lobe findings: Separate meta-analyses of the strong-SM and general-SM effects and a comparison of these two effects.

Volume (mm^3^)	MNI			ALE	Z	Region
	*x*	*y*	*z*			
*Strong-SM*						
1784	-30	-40	-14	0.020		Left fusiform gyrus, PHG and hippocampus
1296	-26	-16	-16	0.016		Left hippocampus, amygdala, and PHG
824	22	-16	-20	0.018		Right PHG and amygdala
328	34	-38	-6	0.015		Right hippocampus
*General-SM*						
4256	-32	-34	-18	0.029		Left PHG and hippocampus
3800	34	-32	-18	0.029		Right PHG and hippocampus
1376	-24	0	-12	0.019		Left lentiform nucleus and amygdala
552	-24	-4	-30	0.013		Left amygdala and hippocampus
168	-28	-50	-10	0.011		Left PHG
*Strong-SM > General-SM*						
432	-28	-32	-6		2.14	Left hippocampus and PHG
*General-SM > Strong-SM*						
1936	30	-28	-18		3.19	Right PHG and hippocampus

ALE, activation likelihood estimation; PHG, parahippocampal gyrus; SM, subsequent memory.

While these findings suggest a potential dissociation in specific regions of the left and right posterior hippocampus/parahippocampal gyrus, the observed laterality pattern is not anticipated by current theories. Furthermore, each observed effect was subtle, particularly in terms of hippocampal involvement, impacting only a restricted portion. Considering these limitations, caution dictates postponing further interpretation until additional research can furnish more robust evidence regarding the reliability and significance of these findings.

The MTL findings suggest that the hippocampus is associated with the strong-SM and general-SM effects to a similar extent, with only minor distinctions involving the posterior hippocampi. To explore this further, a frequency analysis was conducted to assess how many experiments in the strong-SM and general-SM subsamples reported a significant hippocampal effect. The determination of the presence or absence of a significant hippocampal effect relied solely on the judgments of the respective authors, as indicated in their papers. As indicated in Table 3, the proportion of experiments reporting significant hippocampal engagement showed remarkable similarity between the two subsamples: 35.5% in the strong-SM and 33.3% in the general-SM, with no significant statistical difference (χ² < 1). This finding suggests that the strong-SM and general-SM contrasts have comparable sensitivity to encoding-related hippocampal activity.

**Table 3.** Number of experiments reporting significant hippocampal effects and lack thereof in the strong-SM and general-SM subsamples.

		Significant hippocampal effects		
		Present	Absent	Total
Subsample	Strong-SM	11 (35.5%)	20 (64.5%)	31 (100.0%)
	General-SM	12 (33.3%)	24 (66.7%)	36 (100.0%)

### 3.3 Supplementary analyses

This meta-analysis included adults of all ages, operating under the assumption that the neural correlates of encoding may not fundamentally differ across young and older adults. However, a potential concern may arise regarding the appropriateness of analyzing both young and older adults together. To address this issue, supplementary analyses concentrating solely on young adults were conducted, involving 23 experiments in the strong-SM subsample and 31 experiments in the general-SM subsample. The statistical thresholds employed were identical to those for the main analyses.

As presented in Tables S3 and S4 available online, the results of these analyses were highly similar to those observed in the original analyses, although cluster sizes were generally smaller, and some minor clusters fell into non-significance due to lower statistical power. Focusing on the distinctions between the strong-SM and general-SM effects, the left inferior PFC, bilateral inferior frontal junction, left posterior SPL/IPS, left mid-fusiform gyrus, right middle occipital gyrus, and a portion of the left posterior hippocampus/parahippocampal gyrus exhibited a more robust association with the strong-SM effects than the general-SM effects. On the other hand, a portion of the right posterior hippocampus/parahippocampal gyrus displayed the opposite pattern, indicating a stronger association with the general-SM effects than the strong-SM effects.

## 4 Discussion

### 4.1 Main findings

The present meta-analysis yielded the following key findings. First, as outlined in the Introduction, the semantic hypothesis posits that the development of strong memories is associated with elevated activity in the inferior PFC, as opposed to the formation of weaker memories. This hypothesis is supported by the meta-analysis, which demonstrates a more robust association of the bilateral inferior PFC, particularly on the left side, with the strong-SM effects compared to the general-SM effects.

Second, as introduced in the Introduction, the attentional hypothesis suggests that the establishment of robust memories requires heightened activity within the regions comprising the dorsal attention network, as opposed to forming less potent memories. The meta-analysis provides support for this hypothesis by demonstrating that regions exhibiting a stronger association with strong-SM effects, compared to general-SM effects, predominantly correspond to the dorsal attention network. This alignment is evident through the engagement of regions such as the bilateral inferior frontal junction, left posterior SPL/IPL, bilateral mid-fusiform gyrus, and right middle occipital gyrus.

Lastly, the hippocampal hypothesis proposes that the formation of strong memories necessitates increased hippocampal activity, distinct from the formation of weaker memories. However, contrary to this hypothesis, the meta-analysis revealed that the hippocampus exhibited a comparable degree of association with the strong-SM effects as with the general-SM effects, with only minor distinctions involving the posterior hippocampi. Consistent with this finding, the proportion of experiments reporting a significant hippocampal effect remained comparable between the strong-SM and general-SM subsamples, each at approximately one-third.

The lack of evidence does not necessarily indicate the absence of an effect; various reasons could contribute to the absence of evidence for the hippocampal hypothesis. In this context, three factors are being considered to explain the absence of evidence. First, one might posit that the lack of evidence is attributable to fMRI signal distortions and dropouts, potentially more pronounced in MTL compared to other brain regions (Olman et al., 2009). However, this explanation weakens when considering the existence of fMRI tasks that consistently and strongly activate the hippocampus, such as emotional perception (for a meta-analysis, see Sabatinelli et al., 2011), emotional memory encoding (Dahlgren et al., 2020), retrieval of autobiographical memories (Kim, 2012), and recollection-based recognition (Kim, 2010).

Second, some studies (e.g., Ranganath et al., 2004; Yonelinas, 2002) propose a qualitative distinction between recollection-based and familiarity-based recognition. This proposition is closely tied to the hypothesis, suggesting the exclusive association of the hippocampus with recollection but not with familiarity. This perspective is potentially relevant to the lack of evidence because high-confidence responses may involve not only recollection-based but also certain familiarity-based processes. However, it is crucial to emphasize that only a small fraction of familiarity responses is accompanied by high confidence (J. Wixted & Stretch, 2004). Thus, even if one supports the idea that the hippocampus is not implicated in high-confidence familiarity (but see Smith et al., 2011), it is improbable that this factor alone can entirely account for the lack of supporting evidence.

Finally, and perhaps most likely, the absence of evidence may reflect a subtle effect size of the hippocampal effect. A careful review of relevant prior studies aligns with this perspective. As mentioned in the Introduction, only one of these studies (Dennis et al., 2007) reported a significant hippocampal engagement using the same threshold level for both MTL and non-MTL regions. In the other studies, the hippocampal effect was either not statistically significant (François et al., 2018; Gottlieb & Rugg, 2011; Henson et al., 1999; Kim & Cabeza, 2007) or became apparent only when using a lenient statistical threshold, such as SVC (Kirwan et al., 2008; Qin et al., 2011; Shrager et al., 2008; Song, Jeneson, et al., 2011; Song, Wixted, et al., 2011). These nuanced findings may indicate a hippocampal effect, albeit with a small effect size.

### 4.2 A model of encoding-related brain activity

**Fig. 4.**
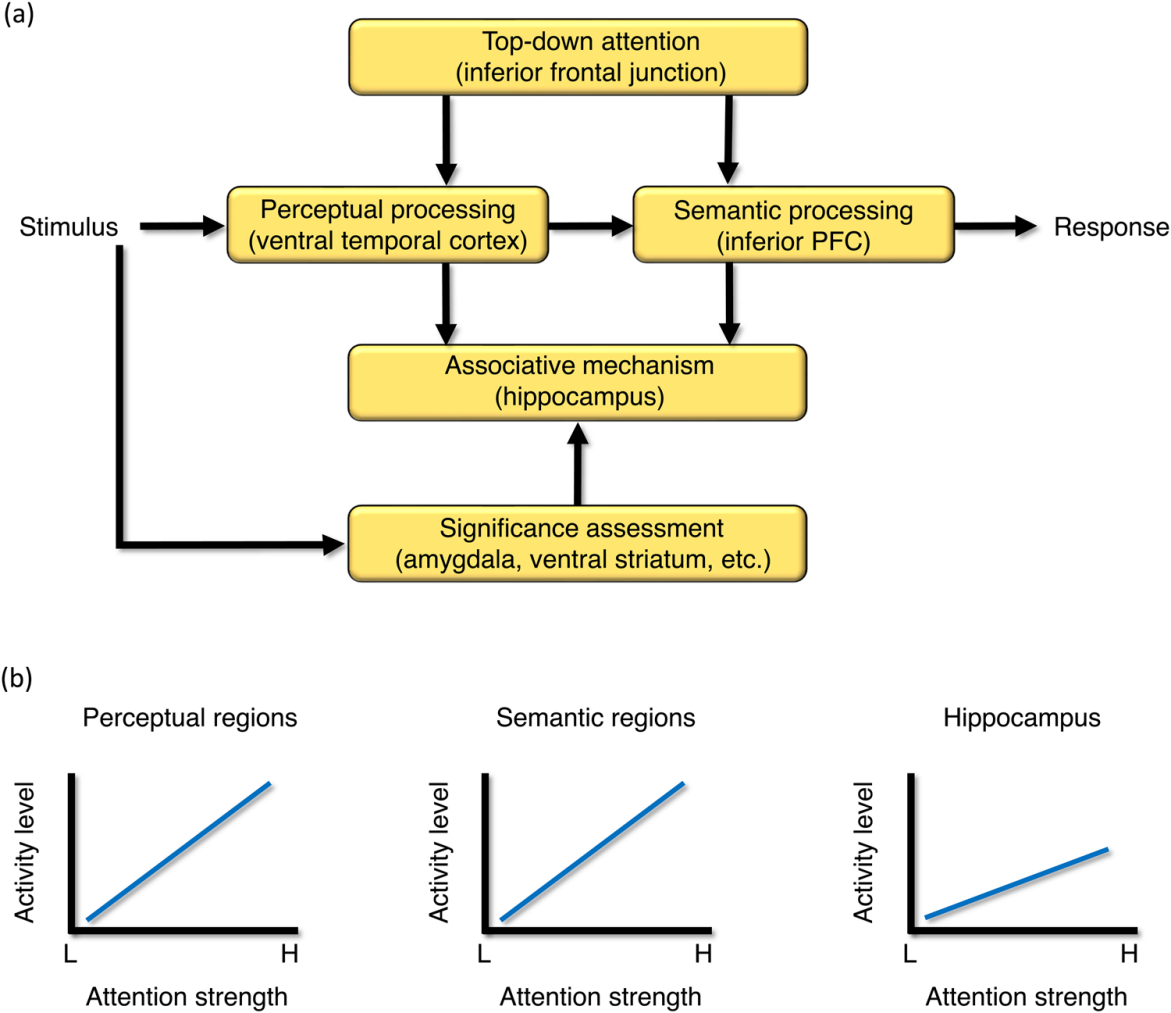
Part (a) presents a schematic representation of the Significance-driven and Attention-driven Memory (SAM) model. Within the model, the top-down attentional mechanism directly influences both perceptual and semantic processing. In contrast, its impact on hippocampal processing occurs indirectly through pathways originating from the perceptual and semantic processing modules. The conveyance of stimulus-significance information to the hippocampus transpires via pathways emerging from the perceptual and semantic processing modules or involves subcortical regions such as the amygdala and ventral striatum. The regions mentioned in parentheses represent the primary areas of interest, though not an exhaustive list of all regions involved. For more comprehensive information, refer to the accompanying text. Part (b) depicts the functional correlation between attention strength and processing activity, as proposed by the SAM model. The model suggests a greater rise in activity in regions linked to perceptual and semantic processing with increased attention focus, contrasting with the hippocampus. H, high; L, low; PFC, prefrontal cortex.

The semantic, attentional, and hippocampal hypotheses underscore distinct brain regions and their specific neural mechanisms. In the following discussion, the author endeavored to synthesize these diverse perspectives into a coherent model of encoding-related brain activity. The model, referred to as "Significance-based and Attention-based Memory (SAM)," was devised to enhance a holistic understanding of the current extensive meta-analytic findings. The broader impetus behind constructing this model was to offer an integrative framework that amalgamates diverse neural and cognitive processes inherent in memory formation into a unified perspective. The existing prevailing viewpoints (Eichenbaum, 2000; Sekeres et al., 2018; Squire et al., 2015; Teyler & Rudy, 2007) endorse the idea or analogous concepts that the process of encoding an experience relies on the reinforcement of connections between hippocampal synapses and the neocortical patterns that become active during that particular experience. This process within the hippocampus is understood to be rooted in associative mechanisms that strengthen the connections among the different elements comprising the experience. Concurrently, intensified processing within the neocortex is believed to solidify the representations within these neocortical regions. The SAM model, depicted schematically in Figure 4a, expands upon this foundational understanding.

#### 4.2.1 Fluctuations in sustained attention

A fundamental source of subsequent memory effects lies in the substantial variability in the encoding performance observed across different trials. According to the SAM model, this trial-to-trial variability is linked to inherent temporal fluctuations in sustained attention (for related views, see de Bettencourt et al., 2018; Kahana et al., 2018; Kim, 2011; Urgolites et al., 2020). This perspective is supported by multiple lines of evidence. First, the changes in sustained attention can be monitored using diverse techniques, including real-time thought sampling, reaction time analysis, and eye movement tracking. By leveraging these methodologies, studies (deBettencourt et al., 2018; Farley et al., 2013; Kahana et al., 2018; Maillet & Rajah, 2014; Thomson et al., 2014) have shown that participants’ attentiveness undergoes continuous oscillations during encoding sessions, mirroring the ebb and flow of attention in everyday situations. Furthermore, fluctuations in attention over time are associated with the likelihood of success or failure of encoding items presented (for a meta-analysis, see Blondé et al., 2022).

Second, as evidenced in both the current investigation and several prior studies, subsequent memory effects (remembered > forgotten) are linked to the dorsal attention network, known for facilitating purposeful attention to incoming information from the external environment. Conversely, as emphasized in meta-analyses conducted by Kim (2011, 2015), subsequent forgetting effects (forgotten > remembered) involve the default mode network, recognized for supporting internal mentation, such as future thinking, mind wandering. The distinctive associations of subsequent memory effects with the dorsal attention network and subsequent forgetting effects with the default mode network align with the perspective that temporal fluctuations in attention play a pivotal role in encoding efficacy and the strength of subsequent memory.

Finally, a growing body of research (e.g., Addante et al., 2015; Otten et al., 2010; Park & Rugg, 2010; Soravia et al., 2016; Urgolites et al., 2020) has substantiated the idea that the brain’s activity just before the onset of a stimulus holds predictive power regarding its subsequent recall. This phenomenon, known as the pre-stimulus subsequent memory effect, underscores that temporal fluctuations in the brain’s endogenous state, rather than stimulus-evoked activity, possess the potential to significantly influence the likelihood of successful encoding. Thus, the pre-stimulus subsequent memory effect aligns with the perspective that trial-to-trial variability in encoding performance can be attributed to inherent temporal fluctuations in sustained attention.

#### 4.2.2 Regional distinctions in attentional impact

The SAM model posits a robust influence of attention on perceptual and semantic processing, with a relatively more subdued effect on hippocampal processing. Previous studies comparing encoding under high and low attention conditions (e.g., full versus divided attention) supported this hypothesis. In these investigations, significant effects consistently emerged within diverse sensory-perceptual regions and the lateral PFC (Kensinger et al., 2003; Uncapher & Rugg, 2005, 2008, 2009; Uncapher et al., 2011), whereas they appeared more rarely within the hippocampus (Uncapher & Rugg, 2008, 2009). The putative regional distinctions in attentional impact explain why the meta-analysis presented compelling evidence to support the attentional and semantic hypotheses while failing to substantiate the hippocampal hypothesis.

The presumed regional variations in attentional influence imply a sharp increase in activity within regions responsible for perceptual and semantic processing, such as the ventral temporal cortex and the inferior PFC, in response to heightened attentional focus. In comparison, the increase in activity is less pronounced in the hippocampus, as shown in Figure 4b. Regarding functional organization, the suggested differences imply a direct impact of top-down attentional mechanisms, facilitated by the inferior frontal junction and its associated regions, on how much perceptual and semantic processing occurs. These mechanisms exert a more indirect influence on hippocampal processing, utilizing the perceptual and semantic processing modules as intermediaries, as shown in Figure 4a. These direct and indirect influences resonate with the subjective experiences. Controlling the intensity of perceptual and conceptual processing is possible by choosing to either focus or not focus on incoming information. On the other hand, people do not possess the same conscious agency over hippocampal encoding (or associative processing) because it operates as a mechanical process beyond conscious awareness.

#### 4.2.3 Attention-driven versus significance-driven encoding

If a purposeful attentional focus does not have a strong but moderate influence on hippocampal processing, what else, if any, has a strong influence? The recently introduced differentiation between attention-driven and significance-driven encoding is instrumental in addressing this issue (Kim, 2022). A key characteristic of attention-driven encoding lies in the deliberate exertion to comprehend or memorize desired information, such as during exam preparation. In contrast, significance-driven encoding is anchored in the view that a biological memory system has evolved to retain survival-significant information (McGaugh, 2013; Miendlarzewska et al., 2016). Significance-driven encoding can produce memories without requiring conscious effort, such as when emotionally charged experiences are etched spontaneously into memory. When experiments use mundane stimuli (as is mostly the case), attention-driven encoding assumes a principal role, while significance-driven encoding occupies a minor role. The SAM model proposes that forming memories within these contexts entails strongly heightened perceptual and semantic processing and moderately elevated hippocampal processing.

In contrast, when survival-relevant stimuli, such as emotional or reward-associated ones, are introduced, significance-driven encoding assumes an equally or even more significant role than attention-driven encoding. The SAM model posits that the formation of memories within such contexts is influenced crucially by strongly escalated hippocampal activity. Substantiating this hypothesis, previous meta-analyses focusing on emotional memory encoding (Dahlgren et al., 2020; Murty et al., 2010) underscored the robust involvement of the hippocampus. Similarly, when encoding was compared between high versus low-reward conditions, a substantial increase in hippocampal activity was consistently observed (Adcock et al., 2006; Krebs et al., 2011; Murty & Adcock, 2013; Wittmann et al., 2005; Wolosin et al., 2012). The conveyance of stimulus-significance information to the hippocampus might occur via routes stemming from the perceptual and semantic processing modules, or it could engage intervening subcortical regions, such as the amygdala and the ventral striatum, as shown in Figure 4a. In summary, the level of hippocampal involvement during encoding tasks may vary according to whether the encoding contexts prioritize attention-driven encoding or significance-driven encoding.

Even if attention has only a moderate impact on hippocampal functioning, as the SAM proposes, an important remaining question is what might be the adaptive advantage of such neurocognitive architecture. As discussed by Kim (2022), while people often remain attentive to ongoing events, only certain significant ones merit long-term storage. Thus, a neurocognitive structure that relies exclusively on attention for encoding is likely maladaptive, potentially leading to an excessively inclusive memory. A more optimal design requires that memory encoding be guided primarily by the adaptive significance of events rather than solely by attention. On the other hand, such a design might have certain disadvantages in exchange for the advantage. For example, many have encountered foreign words being practiced repeatedly, yet they find it challenging to commit them to memory. This difficulty might be linked to the design in which attention alone moderately influences hippocampal processing. These advantages and disadvantages offer additional reasons to emphasize the importance of distinguishing between attention-driven and significance-driven encoding.

#### 4.2.4 Summary

The following summarizes how the SAM model helps explain the current meta-analysis results. First, a fundamental question concerning subsequent memory effects is why activity within certain regions intensifies in specific trials while waning in others, resulting in either successful or unsuccessful encoding. The model links these fluctuations in activity to continuous alterations in sustained attention. Second, the model integrates three distinct perspectives on encoding—semantic elaboration, attentional focus, and hippocampal processing—into a unified framework, facilitating a holistic understanding of the current extensive meta-analysis outcomes. Third, the model proposes that attention exerts a robust influence on perceptual and semantic processing while having a more measured impact on hippocampal processing. These putative differences in the regional impact of attention help explain why the meta-analysis yielded compelling evidence supporting the attentional and semantic hypotheses while failing to substantiate the hippocampal hypothesis. Finally, the model accentuates the heightened involvement of the hippocampus in significance-driven encoding as opposed to attention-driven encoding. This distinct role deepens the comprehension of the lack of evidence for the hippocampal hypothesis, situating it within a more comprehensive context.

In a broader context that extends beyond the scope of the present meta-analyses, the model introduces an integrated framework that synthesizes various encoding-related cognitive and neural processes into a cohesive and unified perspective. The model’s components span a wide range of encoding-related cognitive processes, encompassing perceptual processing, semantic analysis, attentional focusing, significance assessment, and associative mechanisms. A particularly novel aspect is the model’s differentiation between attention-driven and significance-driven encoding, enhancing our understanding of the intricate processes underlying memory formation. To highlight a crucial agenda for future studies, it is essential to recognize that significant stimuli may elicit reactive and heightened attention from participants, potentially blurring the distinctions between attention-driven and significance-driven encoding. Although existing attentional literature provides substantial evidence that the neural correlates of goal-driven and stimulus-driven attention are largely separable (Corbetta & Shulman, 2002; Vossel et al., 2014), further investigations are necessary to explore these distinctions specifically within the context of encoding tasks (Turk-Browne et al., 2013). Initiatives moving in this direction are well-positioned to provide additional insights into the distinctions between attention-driven and significance-driven encoding.

### 4.3 Limitations and summary

This study had several limitations. First, the utilization of neuroimaging meta-analysis represents an inherently coarse methodology, reliant on peak coordinates and encompassing procedurally disparate experiments. Although this study carefully managed certain crucial factors, such as statistical power and the use of verbal and pictorial materials, certain uncontrolled variables may have introduced biases into the results.

Second, this study postulated a connection between the engagement of the inferior PFC and the processing of incoming information at a semantic level. Similarly, it associated the dorsal attention network with focused attention directed towards incoming information. It is essential to note, however, that this study did not directly intervene in semantic processing or attention manipulation; these hypotheses are derived through “reverse inference” (Poldrack, 2006). Therefore, it is imperative to thoroughly scrutinize interpretations of the results based on these hypotheses in future studies.

Lastly, the present meta-analysis and model construction focused exclusively on the outcomes derived from univariate analysis. Nevertheless, an increasing number of studies are now embracing a multivariate approach when analyzing encoding data (e.g., Kuhl et al., 2012; Thakral et al., 2022), which has the potential to offer significant insights. For example, a study by Aly and Turk-Browne (2016) found evidence that the attentional modulation of hippocampal processing is linked to the stability of multivoxel patterns rather than the overall activation. Thus, incorporating outcomes from the pertinent multivariate analysis is essential for the future development of the model.

Within these limitations, this study makes dual contributions to the field of memory encoding, advancing both empirically and theoretically. On the empirical front, this study conducted the first meta-analytic comparison between the strong-SM contrast and general-SM effects. The results revealed that strong-SM effects more robustly engage regions linked to attentional focusing, perceptual processing, and semantic analysis compared to general-SM effects. Conversely, both effects demonstrated a comparable involvement of the hippocampi, with only minor distinctions noted in the posterior hippocampi.

On the theoretical front, this study contributes by introducing a streamlined model of encoding processes, known as the SAM. From a specific perspective, the model’s value lies in its ability to facilitate a holistic understanding of the current meta-analytic findings. In a more comprehensive context, the model introduces an integrated framework that synthesizes various encoding-related cognitive and neural processes into a cohesive and unified perspective. Crucially, it suggests that the neural correlates of encoding vary depending on whether the encoding context prioritizes attention-driven encoding or significance-driven encoding. This nuanced perspective can enhance our understanding of past findings and elevate the sophistication of future hypothesis-driven approaches.

## Supplementary Material

Supplementary Material

## Data Availability

The data supporting the study findings are openly available at https://identifiers.org/neurovault.collection:13389.

## Utilization of Generative AI and AI-Assisted Technologies in the Writing Process

While preparing this manuscript, the author used ChatGPT to improve language and readability. After using this tool, the author reviewed and edited the content as needed and takes full responsibility for the content of the publication.
