# Supplementary material for: Neural and cognitive dynamics leading to the formation of strong memories: A meta-analysis and the SAM model
Source: Imaging Neurosci (Camb). 2024 Feb 22;2:imag-2-00098. doi: 10.1162/imag_a_00098 (PMC12224446; doi:10.1162/imag_a_00098)
Supplement: Supplementary Material [file imag_a_00098-supp.pdf]

Supplementary Material for

Neural and cognitive dynamics leading to the formation of strong memories: A meta-analysis and the SAM model

Hongkeun Kim

Department of Rehabilitation Psychology, Daegu University

Corresponding author: Hongkeun Kim, Ph.D., Department of Rehabilitation Psychology, Daegu University, 201 Daegudae-ro, Gyeongsan-si, Gyeongsangbuk-do, 38453, Republic of Korea, Phone: 053-850-4331, Fax: 053-850-4339, E-mail: hongkn1@gmail.com

Contents

Table S1. Overview of studies included in a meta-analysis addressing the effects of strong-SM presented in the main text

Table S2. Overview of studies included in a meta-analysis addressing the effects of general-SM presented in the main text

Table S3. Analyses limited to experiments with young adults: Non-medial temporal lobe findings in separate meta-analyses of the strong-SM and general-SM effects and a comparison of these two effects

Table S4. Analyses limited to experiments with young adults: Medial temporal lobe findings in separate meta-analyses of the strong-SM and general-SM effects and a comparison of these two effects

Supplementary references: List of all studies utilized in meta-analyses presented in the main text

Table S1. Overview of studies included in a meta-analysis addressing the effects of strong-SM presented in the main text

First author	Year	Subjects	Stimulus category	Encoding task	Retrieval task	Subsequent memory contrast	Foci
Buckner	2001	14	Word	Old/new judgment	Memory confidence rating	HC-hit > miss	13
Clark	2003	17	Word or pseudoword	Syllable judgment	Memory confidence rating	HC-hit > miss	15
Fliessbach	2006	21	Word	Passive viewing	Memory confidence rating	HC-hit > miss	4
Fliessbach	2007	19	Word	Alphabetical, animacy, or size judgment	Memory confidence rating	HC-hit > miss	7
Gordon	2015	66	Word	Living/non-living judgment	Memory confidence rating	HC-hit > miss	3
Gutchess	2005	27	Scene	Water present/absent judgment	Memory confidence rating	HC-hit > miss	17
Kao	2005	16	Scene	Subsequent memory judgment	Memory confidence rating	4 > (2+1)	9
Maril	2011	18	Word	Mental imagining	Memory confidence rating	HC-hit > miss	19
Mei	2010	22	Face	White frame detection	Memory confidence rating	6 > (3+2+1)	13
Morcom	2003	28	Word	Living/non-living judgment	Memory confidence rating	HC-hit > miss	42
Mormino	2012	60	Scene	Water present/absent judgment	Memory confidence rating	HC-hit > miss	23
Murty	2015	20	Object or word	Passive viewing	Memory confidence rating	(6+5) > (3+2+1)	10
Nichols	2006	16	Face	Working memory judgment	Memory confidence rating	4 > (2+1)	8
Oh	2013	15 (Y)	Scene	Water present/absent judgment	Memory confidence rating	HC-hit > miss	15
		23 (PIB-O)	Scene	Water present/absent judgment	Memory confidence rating	HC-hit > miss	12
		13 (PIB+O)	Scene	Water present/absent judgment	Memory confidence rating	HC-hit > miss	9
Otten	2001	17	Word	Syllable judgment	Memory confidence rating	HC-hit > miss	7
Otten	2002	16	Word	Living/non-living judgment	Memory confidence rating	HC-hit > miss	7

Otten	2007	18	Word	Mental imagining	Remember/know decision	Recollected > miss	3
Park	2013	192	Scene	Water present/absent judgment	Memory confidence rating	HC-hit > miss	12
Ranganath	2005	15	Abstract object	Working memory judgment	Memory confidence rating	(6+5) > (1+2)	25
Rizio	2013	24	Word	Directed remembering/forgetting	Remember/know decision	Recollected > miss	15
Rizio	2014	23	Word	Passive viewing	Remember/know decision	Recollected > miss	10
Turk-Browne	2013	31	Scene	Stimulus detection	Memory confidence rating	HC-hit > miss	2
Uncapher	2005	18	Word	Living/non-living judgment	Remember/know decision	Recollected > miss	14
Uncapher	2008	15	Word	Living/non-living judgment	Remember/know decision	Recollected > miss	5
Uncapher	2011	18	Object	Real/unreal object judgment	Memory confidence rating	HC-hit > miss	10
Wagner	1998	13	Word	Abstract/concrete judgment	Memory confidence rating	HC-hit > miss	9
Weis	2004	16	Scene	Building/landscape judgment	Memory confidence rating	HC-hit > miss	7
Yebra	2019	21	Object	Go/no-Go judgment	Remember/know decision	Recollected > miss	8
Zhang	2018	21	Face + Scene	Mental imagining	Memory confidence rating	(6+5) > (3+2+1)	1

HC, high-confidence; PIB-O, Pittsburg compound B negative-old; PIB+O, Pittsburg compound B positive-old; Y, young.

Table S2. Overview of studies included in a meta-analysis addressing the effects of general-SM presented in the main text

First author	Year	Subjects	Stimulus category	Encoding task	Retrieval task	Subsequent memory contrast	Foci
Axmacher	2008	30	Word	Working memory task	Old/new recognition	Hit > miss	13
Baker	2001	18	Word	Abstract/concrete judgment	Old/new recognition	Hit > miss	7
Bastin	2012	17	Word	Silent reading	Old/new recognition	Hit > miss	17
Boenniger	2021	59	Face or scene	Passive viewing	Old/new recognition	Hit > miss	7
Brown	2017	19	Face	Stimulus detection	Old/new recognition	Hit > miss	7
Chee	2003	16	Word	Living/non-living judgment	Memory confidence rating	HC and LC hit > miss	2
Chee	2004	16	Word	Living/non-living judgment	Old/new recognition	Hit > miss	5
Chen	2013	16	Word	Living/non-living judgment	Old/new recognition	Hit > miss	7
Chiu	2015	21	Face	Male/female judgment	Memory confidence rating	HC and LC hit > miss	11
de Zubicaray	2005	14	Word	Passive viewing	Old/new recognition	Hit > miss	6
Dunne	2020	25	Object	Semantic categorization task	Old/new recognition	Hit > miss	4
Elman	2013	19	Scene	People present/absent judgment	Memory confidence rating	HC and LC hit > miss	20
Evans	2017	54	Word	Profession/non-profession-word judgment	Old/new recognition	Hit > miss	1
Evans	2020	32	Word	Profession/non-profession-word judgment	Old/new recognition	Hit > miss	1
Fischer	2007	24	Face (neutral)	Fearful/neutral judgment	Old/new recognition	Hit > miss	2
Fletcher	2003	9	Word	Pleasantness or alphabetical judgment	Old/new recognition	Hit > miss	5

Gold	2006	15	Word	Mental imagining	Item and source retrieval	Item hit > item miss	3
Harvey	2007	12	Scene	People present/absent judgment	Old/new recognition	Hit > miss	4
Henson	2005	22	Word	Alphabetical order judgment	Old/new recognition	Hit > miss	19
Howard	2013	19	Scene + Object	Stimulus detection	Old/new recognition	Hit > miss	4
Jacobs	2015	40	Object	Natural/artificial judgment	Item and source retrieval	Item hit > item miss	14
Jacques	2013	26	Scene	Memory reliving ratings	Memory confidence rating	HC and LC hit > miss	3
Kukolja	2016	40	Object	Natural/artificial judgment	Item and source retrieval	Item hit > item miss	8
Liu	2020	36	Scene	Intentional encoding	Old/new recognition	Hit > miss	19
Qin	2011	20	Scene	Preference ratings	Memory confidence rating	HC and LC hit > miss	1
Quiroz	2015	19	Scene	Warm/cool climate judgment	Old/new recognition	Hit > miss	7
Reber	2002	12	Word	Stimulus detection	Old/new recognition	Hit > miss	4
Reggev	2016	19	Word	Noun/adjective judgment	Memory confidence rating	HC and LC hit > miss	1
Richter	2018	16	Object	Natural/artificial judgment	Old/new recognition	Hit > miss	4
Spencer	2009	12	Object	Natural/artificial judgment	Old/new recognition	Hit > miss	3
Tendorkar	2007	20	Scene	Building present/absent judgment	Item and source retrieval	Item hit > item miss	5
Uncapher	2006	20	Word	Living/non-living judgment	Item and source retrieval	Item hit > item miss	4
Uncapher	2009	15	Object	Animacy and size judgment	Item and source retrieval	Item hit > item miss	10
Weisenbach	2014	23	Word	Silent reading	Old/new recognition	Hit > miss	1
Wimber	2010	20	Word	Syllable counting	Old/new recognition	Hit > miss	27
Yang	2015	26	Word	Abstract/concrete judgment	Memory confidence rating	HC and LC hit > miss	3

HC, high confidence; LC, low confidence

Table S3. Analyses limited to experiments with young adults: Non-medial temporal lobe findings in separate meta-analyses of the strong-SM and general-SM effects and a comparison of these two effects

Lobe	Volume (mm^3)	MNI			ALE	Z	Region
		x	*y*	*z*			
Strong-SM							
Frontal	13496	-42	4	28	0.028		Left inferior PFC and IFJ
	2320	46	8	30	0.019		Right IFJ
Parietal	3000	-28	-76	38	0.017		Left posterior SPL/IPS
Temporal	3928	-46	-54	-14	0.022		Left mid-fusiform gyrus
Occipital	1472	36	-86	0	0.016		Right middle occipital gyrus
General-SM							
Frontal	4712	-46	8	30	0.024		Left IFJ
Temporal	2488	-58	-42	-8	0.015		Left mid-fusiform gyrus and MTG
Strong-SM > General-SM							
Frontal	3816	-46	25	7		3.89	Left inferior PFC
	712	-36	8	32		2.49	Left IFJ
	568	46	16	28		2.66	Right IFJ
Parietal	1776	-30	-75	36		2.72	Left posterior SPL/IPS
Temporal	1760	-52	-54	-20		2.82	Left mid-fusiform gyrus
Occipital	672	40	-84	4		2.53	Right middle occipital gyrus
General-SM > Strong-SM							
(None)							

ALE, activation likelihood estimation; IFJ, inferior frontal junction; IPS, intraparietal sulcus; MTG, middle temporal gyrus; PFC, prefrontal cortex; SM, subsequent memory; SPL, superior parietal lobe.

Table S4. Analyses limited to experiments with young adults: Medial temporal lobe findings in separate meta-analyses of the strong-SM and general-SM effects and a comparison of these two effects

Volume (mm^3)	MNI			ALE	Z	Region
	x	*y*	*z*			
Strong-SM						
1824	-28	-42	-14	0.017		Left fusiform gyrus, PHG and hippocampus
768	-24	-16	-16	0.014		Left amygdala and hippocampus
656	34	-38	-6	0.015		Right hippocampus
208	26	-4	-16	0.011		Right amygdala
General-SM						
2968	-30	-20	-12	0.017		Left hippocampus and PHG
2712	38	-30	-18	0.022		Right PHG and hippocampus
848	-16	-8	-16	0.013		Left amygdala and lentiform nucleus
336	-24	-4	-30	0.013		Left amygdala
232	-28	-50	-10	0.011		Left PHG
Strong-SM > General-SM						
416	-28	-36	-6		2.19	Left hippocampus and PHG
General-SM > Strong-SM						
848	30	-34	-22		2.42	Right PHG and hippocampus

ALE, activation likelihood estimation; PHG, parahippocampal gyrus; SM, subsequent memory.

Supplementary references: List of all studies utilized in meta-analyses presented in the main text

Axmacher, N., Schmitz, D. P., Weinreich, I., Elger, C. E., & Fell, J. (2008). Interaction of working memory and long-term memory in the medial temporal lobe. *Cerebral Cortex, 18*, 2868-2878.

Baker, J. T., Sanders, A. L., Maccotta, L., & Buckner, R. L. (2001). Neural correlates of verbal memory encoding during semantic and structural processing tasks. *NeuroReport, 12*, 1251-1256.

Bastin, C., Feyers, D., Majerus, S., Balteau, E., Degueldre, C., Luxen, A., Maquet, P., Salmon, E., & Collette, F. (2012). The neural substrates of memory suppression: A fMRI exploration of directed forgetting. *PloS one, 7*, e29905.

Boenniger, M. M., Diers, K., Herholz, S. C., Shahid, M., Stöcker, T., Breteler, M. M. B., & Huijbers, W. (2021). A functional MRI paradigm for efficient mapping of memory encoding across sensory conditions. *Frontiers in Human Neuroscience, 14*. 591721.

Brown, T. I., Uncapher, M. R., Chow, T. E., Eberhardt, J. L., & Wagner, A. D. (2017). Cognitive control, attention, and the other race effect in memory. *PloS one, 12*, e0173579.

Buckner, R. L., Wheeler, M. E., & Sheridan, M. A. (2001). Encoding processes during retrieval tasks. *Journal of Cognitive Neuroscience, 13*, 406-415.

Chee, M. W. L., Goh, J. O. S., Lim, Y., Graham, S., & Lee, K. (2004). Recognition memory for studied words is determined by cortical activation differences at encoding but not during retrieval. *NeuroImage, 22*, 1456-1465.

Chee, M. W. L., Westphal, C., Goh, J., Graham, S., & Song, A. W. (2003). Word frequency and subsequent memory effects studied using event-related fMRI. *NeuroImage, 20*, 1042-1051.

Chen, T.-C., Kuo, W.-J., Chiang, M.-C., Tseng, Y.-J., & Lin, Y.-Y. (2013). Over-activation in bilateral superior temporal gyrus correlated with subsequent forgetting effect of Chinese words. *Brain and Language, 126*, 203-207.

Chiu, Y.-C., & Egner, T. (2015). Inhibition-induced forgetting results from resource competition between response inhibition and memory encoding processes. *The Journal of Neuroscience, 35*, 11936-11945.

Clark, D., & Wagner, A. D. (2003). Assembling and encoding word representations: fMRI subsequent memory effects implicate a role for phonological control. *Neuropsychologia, 41*, 304-317.

de Zubicaray, G. I., McMahon, K. L., Eastburn, M. M., Finnigan, S., & Humphreys, M. S. (2005). fMRI evidence of word frequency and strength effects in recognition memory. *Cognitive Brain Research, 24*, 587-598.

Dunne, L., & Opitz, B. (2020). Attention control processes that prioritise task execution may come at the expense of incidental memory encoding. *Brain and Cognition, 144*, 105602.

Elman, J. A., Rosner, Z. A., Cohn-Sheehy, B. I., Cerreta, A. G., & Shimamura, A. P. (2013). Dynamic changes in parietal activation during encoding: Implications for human learning and memory. *NeuroImage, 82*, 44-52.

Evans, S., Dowell, N. G., Tabet, N., King, S. L., Hutton, S. B., & Rusted, J. M. (2017). Disrupted neural activity patterns to novelty and effort in young adult APOE-e4 carriers performing a subsequent memory task. *Brain and Behavior, 7*, e00612.

Evans, S. L., Dowell, N. G., Prowse, F., Tabet, N., King, S. L., & Rusted, J. M. (2020). Mid age APOE ε4 carriers show memory-related functional differences and disrupted structure-function relationships in hippocampal regions. *Scientific Reports, 10*, 3110.

Fischer, H., Sandblom, J., Nyberg, L., Herlitz, A., & Backman, L. (2007). Brain activation while forming memories of fearful and neutral faces in women and men. *Emotion, 7*, 767-773.

Fletcher, P. C., Stephenson, C. M. E., Carpenter, T. A., Donovan, T., & Bullmore, E. T. (2003). Regional brain activations predicting subsequent memory success: An event-related fMRI study of the influence of encoding tasks. *Cortex, 39*, 1009-1026.

Fliessbach, K., Trautner, P., Quesada, C. M., Elger, C. E., & Weber, B. (2007). Cerebellar contributions to episodic memory encoding as revealed

by fMRI. *NeuroImage, 35*, 1330-1337.

Fliessbach, K., Weis, S., Klaver, P., Elger, C. E., & Weber, B. (2006). The effect of word concreteness on recognition memory. *NeuroImage, 32*, 1413-1421.

Gold, J. J., Smith, C. N., Bayley, P. J., Shrager, Y., Brewer, J. B., Stark, C. E. L., Hopkins, R. O., & Squire, L. R. (2006). Item memory, source memory, and the medial temporal lobe: Concordant findings from fMRI and memory-impaired patients. *Proceedings of the National Academy of Sciences, 103*, 9351-9356.

Gordon, B. A., Zacks, J. M., Blazey, T., Benzinger, T. L. S., Morris, J. C., Fagan, A. M., Holtzman, D. M., & Balota, D. A. (2015). Task-evoked fMRI changes in attention networks are associated with preclinical Alzheimer's disease biomarkers. *Neurobiology of Aging, 36*, 1771-1779.

Gutchess, A. H., Welsh, R. C., Hedden, T., Bangert, A., Minear, M., Liu, L. L., & Park, D. C. (2005). Aging and the neural correlates of successful picture encoding: Frontal activations compensate for decreased medial-temporal activity. *Journal of Cognitive Neuroscience, 17*, 84-96.

Harvey, P.-O., Fossati, P., & Lepage, M. (2007). Modulation of memory formation by stimulus content: Specific role of the medial prefrontal cortex in the successful encoding of social pictures. *Journal of Cognitive Neuroscience, 19*, 351-362.

Henson, R. N. A., Hornberger, M., & Rugg, M. D. (2005). Further dissociating the processes involved in recognition memory: An fMRI Study. *Journal of Cognitive Neuroscience, 17*, 1058-1073.

Howard, L. R., Kumaran, D., Ólafsdóttir, H. F., & Spiers, H. J. (2013). Dissociation between dorsal and ventral posterior parietal cortical responses to incidental changes in natural scenes. *PloS one, 8*, e67988.

Jacobs, H. I., Dillen, K. N., Risius, O., Göreci, Y., Onur, O. A., Fink, G. R., & Kukolja, J. (2015). Consolidation in older adults depends upon competition between resting-state networks. *Frontiers in Aging Neuroscience, 6,* 344.

Jacques, P. L. S., Olm, C., & Schacter, D. L. (2013). Neural mechanisms of reactivation-induced updating that enhance and distort memory.

Proceedings of the National Academy of Sciences, 110, 19671-19678.

Kao, Y.-C., Davis, E. S., & Gabrieli, J. D. E. (2005). Neural correlates of actual and predicted memory formation. *Nature Neuroscience, 8*, 1776-1783.

Kukolja, J., Göreci, D. Y., Onur, Ö. A., Riedl, V., & Fink, G. R. (2016). Resting-state fMRI evidence for early episodic memory consolidation: Effects of age. *Neurobiology of Aging, 45*, 197-211.

Liu, Z. X., Rosenbaum, R. S., & Ryan, J. D. (2020). Restricting visual exploration directly impedes neural activity, functional connectivity, and memory. *Cerebral Cortex Communications, 1*, tgaa054.

Maril, A., Avital, R., Reggev, N., Zuckerman, M., Sadeh, T., Sira, L. B., & Livneh, N. (2011). Event congruency and episodic encoding: A developmental fMRI study. *Neuropsychologia, 49*, 3036-3045.

Mei, L., Xue, G., Chen, C., Xue, F., Zhang, M., & Dong, Q. (2010). The “visual word form area” is involved in successful memory encoding of both words and faces. *NeuroImage, 52*, 371-378.

Morcom, A. M., Good, C. D., Frackowiak, R. S. J., & Rugg, M. D. (2003). Age effects on the neural correlates of successful memory encoding. *Brain, 126*, 213-229.

Mormino, E. C., Brandel, M. G., Madison, C. M., Marks, S., Baker, S. L., & Jagust, W. J. (2012). Aβ deposition in aging is associated with increases in brain activation during successful memory encoding. *Cerebral Cortex, 22*, 1813-1823.

Murty, V. P., DuBrow, S., & Davachi, L. (2015). The simple act of choosing influences declarative memory. *The Journal of Neuroscience, 35*, 6255-6264.

Nichols, E. A., Kao, Y. C., Verfaellie, M., & Gabrieli, J. D. (2006). Working memory and long-term memory for faces: Evidence from fMRI and global amnesia for involvement of the medial temporal lobes. *Hippocampus, 16*, 604-616.

Oh, H., & Jagust, W. J. (2013). Frontotemporal network connectivity during memory encoding is increased with aging and disrupted by beta-amyloid. *The Journal of Neuroscience, 33*, 18425-18437.

Otten, L. (2007). Fragments of a larger whole: Retrieval cues constrain observed neural correlates of memory encoding. *Cerebral Cortex, 17*, 2030.

Otten, L. J., Henson, R. N. A., & Rugg, M. D. (2002). State-related and item-related neural correlates of successful memory encoding. *Nature Neuroscience, 5*, 1339-1344.

Otten, L. J., & Rugg, M. D. (2001). Task-dependency of the neural correlates of episodic encoding as measured by fMRI. *Cerebral Cortex, 11*, 1150-1160.

Park, H., Kennedy, K. M., Rodrigue, K. M., Hebrank, A., & Park, D. C. (2013). An fMRI study of episodic encoding across the lifespan: Changes in subsequent memory effects are evident by middle-age. *Neuropsychologia, 51*, 448-456.

Qin, S., van Marle, H. J. F., Hermans, E. J., & Fernández, G. (2011). Subjective sense of memory strength and the objective amount of information accurately remembered are related to distinct neural correlates at encoding. *The Journal of Neuroscience, 31*, 8920-8927.

Quiroz, Y. T., Celone Willment, K., Castrillon, G., Muniz, M., Lopera, F., Budson, A., & Stern, C. E. (2015). Successful scene encoding in presymptomatic early-onset Alzheimer’s disease. *Journal of Alzheimer’s Disease*, *47*, 955-964.

Ranganath, C., Cohen, M. X., & Brozinsky, C. J. (2005). Working memory maintenance contributes to long-term memory formation: Neural and behavioral evidence. *Journal of Cognitive Neuroscience, 17*, 994-1010.

Reber, P. J., Siwiec, R. M., Gitleman, D. R., Parrish, T. B., Mesulam, M.-M., & Paller, K. A. (2002). Neural correlates of successful encoding identified using functional magnetic resonance imaging. *The Journal of Neuroscience, 22*, 9541-9548.

Reggev, N., Bein, O., & Maril, A. (2016). Distinct neural suppression and encoding effects for conceptual novelty and familiarity. *Journal of*

Cognitive Neuroscience, 28, 1455-1470.

Richter, N., Beckers, N., Onur, O. A., Dietlein, M., Tittgemeyer, M., Kracht, L., Neumaier, B., Fink, G. R., & Kukolja, J. (2018). Effect of cholinergic treatment depends on cholinergic integrity in early Alzheimer's disease. *Brain, 141*, 903-915.

Rizio, A. A., & Dennis, N. A. (2013). The neural correlates of cognitive control: Successful remembering and intentional forgetting. *Journal of Cognitive Neuroscience, 25*, 297-312.

Rizio, A. A., & Dennis, N. A. (2014). The cognitive control of memory: Age differences in the neural correlates of successful remembering and intentional forgetting. *PloS one, 9*, e87010.

Spencer, T. J., Montaldi, D., Gong, Q.-Y., Roberts, N., & Mayes, A. R. (2009). Object priming and recognition memory: Dissociable effects in left frontal cortex at encoding. *Neuropsychologia, 47*, 2942-2947.

Tendolkar, I., Arnold, J., Petersson, K. M., Weis, S., Anke, B.-D., van Eijndhoven, P., Buitelaar, J., & Fern?dez, G. (2007). Probing the neural correlates of associative memory formation: A parametrically analyzed event-related functional MRI study. *Brain Research, 1142*, 159-168.

Turk-Browne, N. B., Golomb, J. D., & Chun, M. M. (2013). Complementary attentional components of successful memory encoding. *NeuroImage, 66*, 553-562.

Uncapher, M., & Rugg, M. (2009). Selecting for Memory? The Influence of selective attention on the mnemonic binding of contextual information. *The Journal of Neuroscience, 29*, 8270-8279.

Uncapher, M. R., Hutchinson, J. B., & Wagner, A. D. (2011). Dissociable effects of top-down and bottom-up attention during episodic encoding. *The Journal of Neuroscience, 31*, 12613-12628.

Uncapher, M. R., Otten, L. J., & Rugg, M. D. (2006). Episodic encoding is more than the sum of its parts: An fMRI investigation of multifeatural

contextual encoding. *Neuron, 52*, 547-556.

Uncapher, M. R., & Rugg, M. D. (2005). Encoding and the durability of episodic memory: A functional magnetic resonance imaging study. *The Journal of Neuroscience, 25*, 7260-7267.

Uncapher, M. R., & Rugg, M. D. (2008). Fractionation of the component processes underlying successful episodic encoding: A combined fMRI and divided-attention study. *Journal of Cognitive Neuroscience, 20*, 240-254.

Wagner, A. D., Schacter, D. L., Rotte, M., Koutstaal, W., Maril, A., Dale, A. M., Rosen, B. R., & Buckner, R. L. (1998). Building memories: Remembering and forgetting of verbal experiences as predicted by brain activity. *Science, 281*, 1188-1191.

Weis, S., Klaver, P., Reul, J., Elger, C. E., & Fernandez, G. (2004). Temporal and cerebellar brain regions that support both declarative memory formation and retrieval. *Cerebral Cortex, 14*, 256-267.

Weisenbach, S. L., Kassel, M. T., Rao, J., Weldon, A. L., Avery, E. T., Briceno, E. M., Ajilore, O., Mann, M., Kales, H. C., & Welsh, R. C. (2014). Differential prefrontal and subcortical circuitry engagement during encoding of semantically related words in patients with late-life depression. *International Journal of Geriatric Psychiatry, 29*, 1104-1115.

Wimber, M., Heinze, H.-J., & Richardson-Klavehn, A. (2010). Distinct frontoparietal networks set the stage for later perceptual identification priming and episodic recognition memory. *The Journal of Neuroscience, 30*, 13272-13280.

Yang, H., Cai, Y., Liu, Q., Wang, Q., Zhao, X., Chen, C., & Xue, G. (2015). Differential neural correlates underlie judgment of learning and subsequent memory performance. *Frontiers in Psychology, 6,* 1699.

Yebra, M., Galarza-Vallejo, A., Soto-Leon, V., Gonzalez-Rosa, J. J., de Berker, A. O., Bestmann, S., Oliviero, A., Kroes, M. C. W., & Strange, B. A. (2019). Action boosts episodic memory encoding in humans via engagement of a noradrenergic system. *Nature Communications, 10*, 3534.

Zhang, W., van Ast, V. A., Klumpers, F., Roelofs, K., & Hermans, E. J. (2018). Memory contextualization: The role of prefrontal cortex in functional integration across item and context representational regions. *Journal of Cognitive Neuroscience, 30*, 579-593.
